# Supplementary figures and images for: mRNA 5′ terminal sequences drive 200-fold differences in expression through effects on synthesis, translation and decay
Source: PLoS Genet. 2022 Nov 28;18(11):e1010532. doi: 10.1371/journal.pgen.1010532 (PMC9731452; doi:10.1371/journal.pgen.1010532)

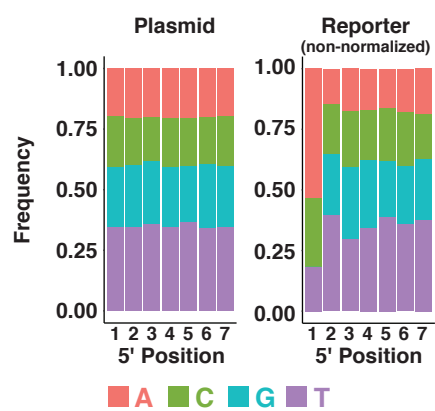

Supplement: S1 Fig — Sequencing libraries prepared from 5pseq plasmid or HeLa cells stably expressing the 5pseq library were analyzed to determine nucleotide frequencies in the first 7 nt of the expected (plasmid) or expressed (from cells) mRNA, respectively. (PDF) [file pgen.1010532.s001.pdf]

**A**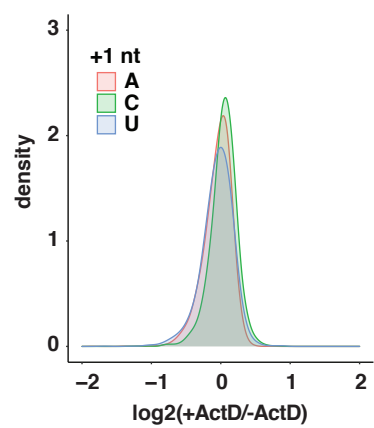**B**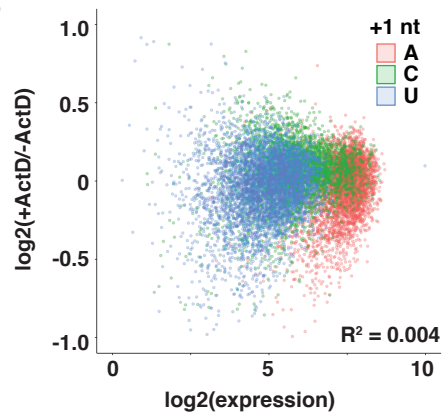

Supplement: S2 Fig — The stabilities of 5pseq library mRNAs are similar under control conditions (A) Decay rates of library mRNAs are similar under control conditions. HeLa cells expressing library mRNAs were treated with 2 μg/mL Actinomycin D for 0 or 2 h in 2 biological replicates. Libraries prepared from extracted mRNA were analyzed to determine relative changes in levels between ActD-treated (ActD+) and untreated (ActD-) conditions. Log2 changes in levels are plotted separately for mRNAs initiating with the indicated nucleotide. (B) Expression levels of library mRNAs are not correlated with decay rates. Decay rates (ActD+/ActD-) from (A) are compared with expression levels of library mRNAs from (Fig 2A) expressed in HeLa cells. (PDF) [file pgen.1010532.s002.pdf]

Control-treated

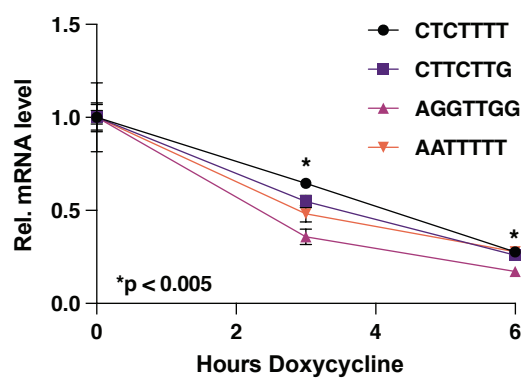

Torin 1-treated

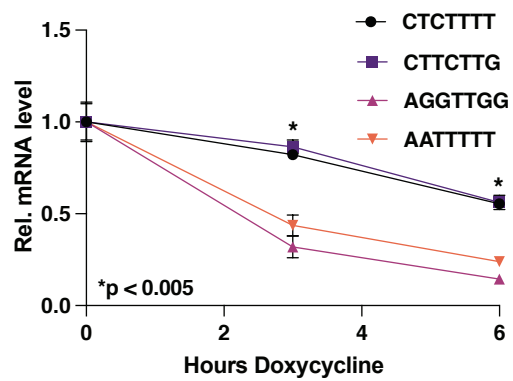

Supplement: S3 Fig — HEK-293T cells were transfected with doxycycline-repressible plasmids encoding 5pseq library mRNAs with the indicated 5′ sequences. Cells were then treated with vehicle (DMSO) or 250 nM Torin 1 and 1 μg/mL doxycycline for the indicated times. mRNA levels were analyzed by qPCR (n = 3, significance by t-test). (PDF) [file pgen.1010532.s003.pdf]
